# Orofacial Pain and Temporomandibular Disorders Education at Umm Al-Qura University: Perceptions and Curriculum Improvement Recommendations

**DOI:** 10.3390/dj13100465

**Published:** 2025-10-10

**Authors:** Mohammad Hasan Al-Harthy

**Affiliations:** Department of Basic and Clinical Oral Sciences, Faculty of Dental Medicine, Umm Al-Qura University, Makkah 21955, Saudi Arabia; mhharthy@uqu.edu.sa

**Keywords:** orofacial pain, temporomandibular disorders, dental education, curriculum evaluation

## Abstract

**Objectives:** To evaluate dental students’ and recent graduates’ perceptions of the integration, effectiveness, quality, and clinical relevance of orofacial pain (OFP) and temporomandibular disorders (TMDs) education in the Oral Medicine (OM) course at Umm Al-Qura University’s Faculty of Dental Medicine (UQUDENT), and to identify educational gaps and opportunities for curriculum improvement. **Methods:** This cross-sectional study was conducted using a self-administered online questionnaire distributed via Google Forms to 117 participants, including fourth- to sixth-year students, interns, and recent (2022–2024) graduates. Respondents provided demographic information and assessed the effectiveness (10 items), quality (4 items), and value/relevance (4 items) of the OM course using a 5-point Likert scale. **Results:** Respondents provided moderate ratings for course effectiveness (mean = 35.2/50) and quality (mean = 13.5/20), and rated OFP/TMD content as having high clinical value (mean = 16.1/20). They had limited confidence in OFP/TMD diagnosis (mean = 3.09/5) and management (mean = 3.19/5). More than 80% believed the curriculum should include more OFP/TMD content. No significant differences were observed by gender, sector, study/work area, clinical exposure (all *p* > 0.05). **Conclusions:** Students recognize the importance of OFP/TMD education, but the current curriculum may be insufficiently structured to build competence. Improvement of curricular depth, teaching methods, and clinical exposure is recommended.

## 1. Introduction

Orofacial pain (OFP) is caused by a range of conditions affecting the nerves, muscles, and joints of the mouth and face, including temporomandibular disorders (TMDs). An estimated 10–15% of people worldwide have TMDs and OFP [1]. Although these conditions necessitating accurate diagnosis and management requiring specialized expertise. Several studies have shown that general dental professionals lack the required knowledge [2,3,4,5].

To effectively address these circumstances, and as incorrect diagnosis or insufficient care could result in long-term pain and reduced quality of life, a strategy that combines education with hands-on experience is needed [6]. These subjects should be prioritized in dental education to adequately prepare professionals to handle these intricate conditions. The information acquired by dental students throughout their undergraduate training impacts their ability to diagnose conditions and formulate suitable treatment plans [5].

In many dental education programs, OFP and TMDs are covered under the Oral Medicine (OM) specialty, which deals with the non-surgical treatment of oral and facial diseases [7]. Education about OFP and TMDs in OM courses has recently improved significantly with curricular changes, the use of competency-based frameworks, and the inclusion of evidence-based practices and teaching strategies [8]. This educational content has become especially important considering the growing demand for dental professionals to effectively handle OFP and TMD cases. Reflecting the divide between theoretical knowledge and clinical preparedness, research has shown that students typically believe that they do not have sufficient knowledge to diagnose and cure these conditions [9]. A recent review revealed significant variation in teaching approaches, with some courses emphasizing occlusal models rather than biopsychosocial frameworks, and thus, providing inadequate or out-of-date training. The lack of specialized teachers and poor student understanding have been shown to lead to poor educational outcomes in this field (54% average student accuracy) [10]. Another study highlighted the lack of self-esteem among dental practitioners regarding their ability to treat chronic OFP, also pointing to the need for better education and skill development [11].

A qualitative study conducted by Schiekirka et al. [12] in 2012 revealed that students perceive evaluations as instruments for the enhancement of medical education. The authors indicated that various factors, including instructional content and processes, student and teacher characteristics, and educational outcomes, influence the quality of instruction [12]. Moreover, students who favored online assessments expressed that the dissemination of findings among faculty and peers to foster improvement would be highly valued [12]. Student feedback can pinpoint areas in which improvement is needed, such as teachers’ lack of experience or unclear guidance [13].

Umm Al Qura University’s Faculty of Dental Medicine (UQUDENT) has a Bachelor of Dental Surgery program and developed its existing dental curriculum with emphasis on integration in alignment with international standards [14]. This curriculum covers 6 years, followed by a compulsory year of internship. The initial year serves as a preparatory phase; in the second through sixth years, 39 general and dental courses comprising a total of 323 credit hours are delivered [14]. The OM course at UQUDENT, with 4.0 credits each semester (3.0 credits theoretical and 1.0 credit clinical), is delivered in the department of Basic and Clinical Oral Sciences. This clinical course aims to familiarize students with the diagnosis and management of diseases of soft tissues of the head and neck, as well as the systemic effects of these diseases, focusing on systemic diseases with oral manifestations and/or implications for dental treatment. Additionally, students learn about the differential diagnosis and management of common oral lesions, such as white lesions, red lesions, blood disorders, and ulcerative and vesiculobullous diseases. The course also covers topics such as allergies, basic immunity, dental emergencies, special care dentistry, halitosis, xerostomia, OFP, and TMDs.

This study was conducted to evaluate dental students’ and graduates’ perceptions of the integration and quality of OFP and TMD education in UQUDENT’s OM course, with an emphasis on identifying gaps and opportunities for curriculum improvement. It is considered as a step toward enhancing the quality of education in this area and its alignment with the changing demands and standards of patient care.

## 2. Materials and Methods

### 2.1. Design and Participants

A cross-sectional design and questionnaire were used in this study to assess UQUDENT students’ perceptions of OFP and TMD education, encompassing the course material, teaching techniques, and students’ preparedness to deal with these conditions. Dental students in the clinical (fourth through sixth) years of the program, interns, and graduates from the 2022–2024 classes participated. Participation was voluntary, and a total of 117 individuals (57 students, 20 interns, and 40 graduates) filled out the questionnaire. The study was approved by the Institutional Review Board (IRB) at UQU (IRB no. HAPO-02-K-012-2025-05-2699).

### 2.2. Data Collection

Data were collected using a self-administered anonymous questionnaire adopted with modification from Al-Ansari and Nazir [15], which was distributed online via email and Google Forms. Since no fully validated questionnaire was available for this specific context, we took the crucial further step of face-validating our modified questionnaire. To accomplish this, two senior faculty members—a professor in public health and an assistant professor and consultant in OFP and TMDs—reviewed it independently. They examined everything from the relevance of the content and the readability of the language to the spelling, grammar, and overall appropriateness for our target population. Based on their input, a few minor adjustments were made, ensuring the final version was linguistically correct, culturally suitable, and exactly aligned with this research objectives.

The questionnaire had three main sections addressing distinct thematic areas. Section A (Demographic Information) was used to collect baseline data for the contextualization of respondents’ perspectives. It included questions related to respondents’ gender, age, academic or professional status (e.g., student, intern, graduate), and current study or workplace (governmental vs. private). Additionally, participants were asked whether they had prior clinical exposure to OFP and TMD cases, which was essential for the correlation of their perceptions with their actual experience in the field. Section B (Effectiveness of OFP/TMD Teaching) assessed the educational impact of the OFP and TMD components of the OM course. Using a 5-point Likert scale (1, strongly disagree; 5, strongly agree), participants rated the perceived effectiveness of the course content across several domains. These included the relevance of the topics to clinical practice and their contributions to theoretical knowledge, clinical skills, decision making, patient outcomes, professional motivation, and collaboration with peers. This section enabled us to measure cognitive and practical dimensions of learning outcomes associated with the OFP/TMD curriculum. Section C (Course Quality and Future Needs) explored participants’ overall satisfaction with the course structure and content delivery, in addition to their perceived readiness to manage OFP and TMD cases. Items assessed the clarity of instructors’ explanations, the effectiveness of teaching methods, and the perceived need to expand OFP/TMD training in future curricula. Additionally, participants were asked about their confidence in their ability to diagnose and manage OFP and TMDs after course completion and their views on the importance of these competencies for professional licensure. This section provided insight into current educational gaps and suggestions for curriculum development.

All qualified students and recent graduates of the UQUDENT were invited to participate, following the CHERRIES checklist for online survey reporting. The target population totaled 72 fourth-year students, 56 fifth-year students, 49 sixth-year students, 43 interns, and 158 recent graduates (total eligible: 378). Invitations were sent via official institutional email. Over the 12-week survey window (May–July 2025), two reminder communications were distributed. Participation was anonymous and voluntary, with electronic consent obtained at the beginning of the questionnaire in Google Form format, and no benefits were offered as shown in Supplementary file S1. To ensure the feedback was honest and unfiltered, robust measures were implemented to prevent courtesy bias. The UQUDENT academic office independently managed all communications, and to eliminate any influence from grading, data were collected only after final course grades were released. Participation remained strictly voluntary and anonymous; for instance, the Google Form was configured to block the gathering of IP addresses and emails. These essential safeguards reduced the likelihood of responses being swayed by the instructor’s role, thereby supporting the validity of the findings.

A total of 117 participants completed the questionnaire, yielding an aggregate response rate of 30.9%. Rather than an a priori power calculation, the questionnaire aimed at the whole reachable population (i.e., a census) of fourth- to sixth-year students, interns, and recent graduates (2020–2024) to guarantee full institutional representation. The full dataset is available in Supplementary Table S2.

### 2.3. Statistical Analysis

The statistical analyses were performed using SPSS (version 28.0; IBM Corporation, Armonk, NY, USA). Descriptive statistics [counts, frequencies, percentages, means, and standard deviations (SDs)] are employed for data summarization. The *t* test and analysis of variance were performed for the statistical tests, with the significance level set at *p* < 0.05.

## 3. Results

A total of 117 responses were obtained, yielding a total response rate of 30.9%. For fourth-year students, stratified response rates were 34.7% (25/72); for fifth-year students, they were 28.6% (16/56); for sixth-year students, they were 30.6% (15/49); for interns, they were 27.9% (12/43); and for recent graduates, they were 30.4% (49/158).

The demographic data regarding the participants’ gender, age, sector, current study/workplace, and previous exposure to training in orofacial pain or TMDs is shown in Table 1.

The participants were asked 10 questions about the effectiveness of the course, to which they answered as shown in Table 2. Means were calculated in a continuous manner using a Likert scale, where 1 indicates “strongly disagree” and 5 indicates “strongly agree”. Higher scores reflect stronger agreement, while lower scores indicate stronger disagreement. When the scores of all 10 questions were summed, a total mean of 24.79 (SD = 9.91) out of 50 points was obtained. 

The participants evaluated the course quality in four questions, and their answers are shown in Table 3. Means were calculated in a continuous manner using the Likert scale, where 1 indicates “strongly disagree” and 5 indicates “strongly agree”. When the scores of all 4 questions were summed, a total mean of 10.53 (SD = 4.2) out of 20 was obtained.

The participants answered four questions related to the value of the course and its relevance to the clinical practice, as shown in Table 4. Means were calculated in a continuous manner using the Likert scale, where 1 indicates “strongly disagree” and 5 indicates “strongly agree”. When the scores of all 4 questions were summed, a total mean of 7.95 (SD = 3.11) was obtained.

The total scores for effectiveness, course quality, and course value were analyzed against gender, sector, study/work area, and previous clinical exposure. Initial assessment of normality using the Shapiro–Wilk test indicated significant deviations from normality (*p* < 0.05). Accordingly, nonparametric tests (Mann–Whitney U and Kruskal–Wallis) were applied for sensitivity analyses, as shown in Table 5. Nevertheless, as most subgroups had sufficiently large sample sizes (n > 30), the Central Limit Theorem justifies the use of parametric tests (independent-samples *t*-test and one-way ANOVA), as the sampling distribution of the mean approximates normality under such conditions. Therefore, parametric tests were used in addition to non-parametric tests for sensitivity analyses. Total scores were calculated by summing responses on the Likert scale, with higher totals reflecting stronger agreement and lower totals reflecting stronger disagreement. Both parametric and nonparametric tests yielded consistent results, with no statistically significant differences observed (all *p*-values > 0.05), as detailed in Table 5.

## 4. Discussion

This study was conducted to examine the perceptions of dental students and recent graduates (i.e., within 3 years since graduation) regarding the OFP/TMD education they received in the OM course at a governmental university in Saudi Arabia. Though the response rate was low, the demographic distribution of respondents closely matches that of the eligible population, hence reducing the possibility of systematic nonresponse bias.

The results highlight notable gaps between curriculum delivery and the participants’ clinical competence, as many participants reported low confidence in their ability to independently diagnose and manage these conditions. The findings reflect moderate degrees of perceived course effectiveness and quality; the participants demonstrated strong recognition of the clinical importance of OFP and TMDs and reported the need for the enhancement of their curricular coverage. These findings align with national and international observations that many undergraduate dental curricula provide insufficient structured exposure, creating a knowledge–confidence gap among new graduates [2,16,17].

The moderate overall course effectiveness score and participants’ limited confidence in their diagnostic abilities reflect a persistent gap in practical exposure. Consistent with these findings, previous studies conducted in Saudi Arabia have shown that newly graduated dentists struggle with OFP and TMD diagnosis compared with specialists [16]. Internationally, similar deficiencies in the integration of OFP and TMD content into predoctoral curricula have been reported [17,18]. Together, these findings suggest that systemic and curricular reforms are required to bridge this gap.

The participants’ perceptions of the quality of the OFP and TMD content and the associated teaching methods also point to the need for attention in these areas. Participants reported that the course content was valuable, but many expressed dissatisfaction with the clarity of instruction and explanation, suggesting a need for improved content delivery methods. Similar concerns have been raised in previous evaluations of OM curricula, both in the region and internationally; the lack of well-structured, competency-based modules has been found to limit students’ ability to apply the theoretical knowledge that they have gained [4,16,19].

Another key observation from this study is the lack of a significant difference in scores based on respondents’ gender, institution type, or previous exposure to OFP and TMD cases. This finding suggests that the challenges of OFP/TMD education are systemic, rather than subgroup-specific. It underscores the need for national-level curricular reform, such as the harmonization of OM course objectives across Saudi dental colleges and the embedding of OFP and TMD competencies in the OM curriculum through the Saudi Commission for Health Specialties (SCFHS). In 2020, the SCFHS acknowledged OFP as a distinct dental specialty in Saudi Arabia [16]. Currently, no separate undergraduate-level course in OFP/TMD exists; however, UQUDENT introduced a 3-year OM fellowship with a core course in 2024.

International models provide a useful transition benchmark. OFP was formally acknowledged by the American Dental Association (ADA) as a dental specialty in March 2020, prompting educational authorities to require the integration of OFP and TMD education into all predoctoral dental curricula [20]. The Commission on Dental Accreditation guidelines focus on OFP-related skills, but TMD-related curriculum requirements remain somewhat lacking [20]. On the other hand, competency-based frameworks, such as those proposed by the American Academy of Orofacial Pain (AAOP) and recently adopted in several European and North American dental programs, have led to measurable improvements in students’ confidence and clinical decision making [8,19]. A survey conducted to investigate the existing range of topics, teaching methods, and coverage of TMD coursework in dental programs for predoctoral students across the United States revealed that guidelines for TMD education differed greatly between the programs examined, and that newer teaching plans had more hours, covered a wider range of topics, and provided more chances for patient involvement [21].

Despite regional differences, the consensus in the literature clearly indicates that more attention to OFP and TMDs is needed in dental curricula. A comprehensive review conducted by Costa et al. [11] revealed that OFP education is necessary to improve patient care and should cover core competencies in pain diagnosis and management for all dentists. Unfortunately, many undergraduate dental curricula (including that of UQUDENT, until recently) have not kept pace with the evolving understanding of OFP and TMDs. The result is a generation of dentists who may lack the confidence and skills to manage common pain disorders. The findings reinforce reports from Saudi Arabia and other countries indicating that insufficient training in OFP and TMDs at the predoctoral level leaves practitioners feeling unprepared and may contribute to suboptimal patient outcomes [16].

The current study’s findings highlight the importance of aligning local curricula with international standards. The Diagnostic Criteria for TMD (DC/TMD) provides standardized diagnostic criteria that should be adopted earlier in training to enhance diagnostic competencies [22]. Likewise, the AAOP has outlined guidelines that specify competencies for the assessment and management of OFP in dental curricula [6]. The International Association for the Study of Pain (IASP) core curriculum outlines structured recommendations for integrating pain education across disciplines, including dentistry [23]. Although these frameworks offer recommendations rather than official accreditation criteria, they are still useful and can be considered as benchmarks for guiding curriculum development and placing Saudi dental education within an international context.

### 4.1. Strengths and Limitations

This study is the first to specifically evaluate dental students’ perspectives on the OFP/TMD content in UQUDENT’s dental curriculum. It adds valuable local data to the global discussion on OFP education. Another strength is the inclusion of students at different levels (undergraduates, interns, and recent graduates), which provides a comprehensive view of how OFP/TMD training is perceived across the educational continuum. The use of a validated questionnaire covering multiple domains (knowledge, skills, decision making, and course quality) is another strength, as it provided detailed insights into specific weaknesses of the curriculum (e.g., instruction clarity, content organization, and clinical relevance).

However, several limitations of the study must be acknowledged. First, it focused on a single institution’s curriculum, which may limit the generalizability of the findings to other dental schools in Saudi Arabia and beyond. Dental curricula vary between universities; for example, the results may not reflect the situation at institutions with dedicated OFP/TMD courses and those that provide more extensive clinical training. Second, the self-reported perceptions of students and recent graduates were collected, which can be influenced by individual expectations or recall bias. We did not directly measure participants’ objective knowledge or competence in OFP/TMD, and some respondents may have under- or over-rated their abilities. Nonetheless, the subjective confidence and satisfaction levels are important educational outcomes, as they can influence the willingness to treat patients with OFP/TMD or pursue further training. Another potential limitation is that subtopics within OFP/TMD education were not distinguished; students may have received adequate instruction in certain areas (e.g., basic TMD anatomy) but not others (e.g., chronic pain management or multidisciplinary care). A more granular analysis of subtopics could be useful in future research to pinpoint the most deficient content areas. Furthermore, certain questions—such as “The OFP/TMD content increased my clinical practice”—might be read in various ways (e.g., viewed as enhancement in confidence or skills). Therefore, these findings ought to be interpreted carefully as they may represent the subjects’ impressions rather than their actual clinical performance. To validate and supplement these results, future multi-institutional studies employing mixed-methods designs including objective tests are advised.

While the questionnaire underwent face validation for clarity and relevance by two senior faculty members, it lacks full psychometric validation (e.g., construct validity, factor analysis, or test–retest reliability). Consequently, the findings are best limited to the specific context of the present study. Future research should prioritize a more rigorous and comprehensive validation before this instrument can be used or generalized elsewhere.

Finally, no control or comparison group from another institution or country was included in the study. Such comparison could have strengthened the conclusions by illustrating whether the observed dissatisfaction is a universal phenomenon or specific to our setting. Despite these limitations, the consistency of the findings with those of other studies suggests that the issues identified are not isolated and warrant broader attention.

### 4.2. Recommendations for Curriculum Improvement

#### 4.2.1. Enhancing the Dental Curriculum on OFP and TMDs

The results of this study point to clear opportunities for enhancement of the dental curriculum on OFP and TMDs. Foremost, there is a need to increase the depth of OFP/TMD content in the undergraduate program in any dental school. More than 80% of respondents expressed a desire for more instruction in this area (as reflected by their agreement that content should be increased and is important for practice). It is recommended that a stand-alone OFP/TMD module or course is introduced, rather than solely relying on scattered lectures in other courses to deliver this content. A dedicated course would provide more systematic coverage of core topics, including the basic science of pain, the diagnosis of TMDs and neuropathic pain conditions, the biopsychosocial aspects of chronic pain, and evidence-based management strategies (ranging from occlusal appliance use and physiotherapy to pharmacotherapy and referral). This recommendation is in line with international shifts toward the use of standardized curricula; for example, the AAOP has developed a competency-based core curriculum for predoctoral OFP/TMD education to ensure that all graduates attain fundamental knowledge and skills in this field [19]. The adoption or adaptation of such a core curriculum—in the UQUDENT program specifically and in other dental schools generally—would provide a framework for what students should know upon graduation, ensuring that important topics are not omitted.

#### 4.2.2. Enhancing Clinical Exposure

The students participating in this study did not feel that the theoretical OFP/TMD content translated into improved clinical skills or patient care. To address this issue, the curriculum should include more practical opportunities for students, such as clinical rotations in OFP/TMD clinics. If a full clinical rotation of this type is not feasible, case-based learning and simulation could be implemented. For instance, students could participate in problem-based learning sessions with OFP/TMD case scenarios, or simulations of patient assessment, diagnosis, and treatment planning. Additionally, the encouragement of elective rotations or internships in OFP/TMD could provide deeper exposure for interested students and possibly inspire some of them to pursue specialization in this field, which would ultimately build the national capacity to manage these conditions.

#### 4.2.3. Updating of Teaching Methods

Another recommendation is the updating or modernization of the teaching methods used in OFP/TMD education. Traditional lecture-based delivery may not be sufficient for this topic, which requires the application of strong clinical reasoning and patient communication skills. Interactive teaching methods such as small group discussions, hands-on workshops on masticatory muscle and temporomandibular joint examination, differential diagnoses, radiological image interpretation, and the demonstration of occlusal appliance use could greatly enhance students’ understanding. The use of multimedia resources, including pain assessment videos and online modules (e.g., on the pain education recommendations of the IASP [23]), would complement classroom learning. Given that many participants in this study found the instructors’ explanations to be unclear or the educational content to be poorly organized, faculty development is also important. Faculty training in the latest OFP/TMD evidence and effective pedagogical strategies would help to improve the delivery of content. Where possible, the incorporation of (even simulated) patient encounters into the curriculum could reinforce the relevance of the material, as students who see the real-world impacts of OFP and TMD are more likely to value and retain what they learn [24].

#### 4.2.4. Assessment of Learning Outcomes

Finally, formal assessment of learning outcomes regarding the topic of OFP and TMD would be beneficial. The introduction of specific competency assessments, such as an objective structured clinical examination station on OFP/TMD diagnosis or case presentations, would ensure that students attain a minimum standard of knowledge and skills. A few OFP/TMD case presentations were assigned to some student groups in the UQUDENT OM course for the first time at the end of the last academic year, and this is considered a very good step. Such presentations not only motivate students to engage with the material but also enable the identification of areas in which teaching may need to be reinforced. Regular feedback from students should be sought as the curriculum is revised, with their perspectives helping to guide the iterative improvement of content and teaching approaches.

## 5. Conclusions

In summary, the present study provides valuable insights into the current status of OFP/TMD education in the OM course at UQUDENT. The findings highlight the strengths of the program and specific areas in which improvement is needed; in particular, revealing that the current OFP/TMD curriculum is perceived to insufficiently prepare students for clinical practice. Students reported gaps in knowledge, low confidence in their ability to manage OFP/TMD cases, and a lack of emphasis on these topics in their training.

These results are in line with findings from Saudi Arabia and other countries, which point to the need for enhanced OFP/TMD education. The strengthening of the curriculum through content expansion, the improvement of teaching quality, and increased clinical exposure is paramount. By addressing these deficiencies, UQUDENT could produce dentists who are more competent and confident in the diagnosis and treatment of TMD and other conditions causing OFP. This is expected to ultimately translate into better patient care, as general practitioners form the frontline of the management of these conditions. The findings of this study reflect perceptions from one institution and should be interpreted with caution. National curricular recommendations are framed as proposals for future research.

The momentum seen globally, such as the integration of OFP/TMD content into dental school accreditation standards and the development of core curricula, should be leveraged and adapted locally. Curriculum committees and educational policymakers in our region prioritize OFP/TMD education as a core component of dental training are recommended. The closing of gaps in OFP/TMD education is expected to better equip new dentists to meet the challenges faced in practice, reduce the risk of mismanagement, and improve outcomes for patients with these often-debilitating conditions. The improvements made in response to the findings of this study could serve as a model for other institutions seeking to enhance their dental curricula and ensure that their graduates are well-rounded, competent healthcare professionals.

## Figures and Tables

**Table 1:** Participants’ demographic data.

		*n*	%
Gender	Male	59	50.43
	Female	58	49.57
Age	20–30	116	99.15
	31–40	1	0.85
Study/work area	Governmental dental college	77	65.81
	Governmental workplace	14	11.97
	Private workplace	26	22.22
Current study level or workplace	4th year	20	17.09
	5th year	17	14.53
	6th year	20	17.09
	Intern	20	17.09
	Graduate	40	34.19
Had clinical exposure to orofacial pain or TMD cases as part of your training?	Yes	97	82.91
	No	20	17.09

**Table 2:** The effectiveness of the orofacial pain and TMD course.

	Statement	Disagree n (%)	Neutral *n* (%)	Agree *n* (%)	Total *n* (%)	m (SD) *
1	The Orofacial Pain and TMD content improved my dental knowledge.	84 (71.79)	24 (20.51)	9 (7.69)	117 (100)	2.09 (1.04)
2	The Orofacial Pain and TMD content improved my clinical skills.	75 (64.10)	28 (23.93)	14 (11.97)	117 (100)	2.32 (1.07)
3	The Orofacial Pain and TMD content improved my clinical decision-making.	65 (55.56)	35 (29.91)	17 (14.53)	117 (100)	2.47 (1.07)
4	The Orofacial Pain and TMD content helped me achieve patient satisfaction.	55 (47.01)	44 (37.61)	18 (15.38)	117 (100)	2.57 (1.09)
5	The Orofacial Pain and TMD content increased my clinical practice.	57 (48.72)	32 (27.35)	28 (23.93)	117 (100)	2.62 (1.17)
6	The Orofacial Pain and TMD content improved the quality of dental care provided to my patients.	69 (58.97)	32 (27.35)	16 (13.68)	117 (100)	2.34 (1.11)
7	The Orofacial Pain and TMD content improved my motivation for learning.	71 (60.68)	32 (27.35)	14 (11.97)	117 (100)	2.32 (1.07)
8	The Orofacial Pain and TMD content helped me network with professionals in dentistry.	72 (61.54)	29 (24.79)	16 (13.68)	117 (100)	2.34 (1.11)
9	I am confident to diagnose orofacial pain and TMD after taking the Oral Medicine course.	46 (39.32)	30 (25.64)	41 (35.04)	117 (100)	2.91 (1.29)
10	I am confident to manage patients with orofacial pain and TMD after taking the Oral Medicine course.	50 (42.74)	35 (29.91)	32 (27.35)	117 (100)	2.81 (1.25)

* Higher scores reflect stronger agreement, while lower scores indicate stronger disagreement.

**Table 3:** Participants’ evaluation of the quality of the orofacial pain and TMD course.

	Statement	Disagree *n* (%)	Neutral *n* (%)	Agree *n* (%)	Total *n* (%)	m (SD) *
1	I am satisfied with the quality of the Orofacial Pain and TMD content in the Oral Medicine course	49 (41.88)	33 (28.21)	35 (29.91)	117 (100)	2.8 (1.22)
2	The Orofacial Pain and TMD content were effectively organized.	55 (47.01)	33 (28.21)	29 (24.79)	117 (100)	2.66 (1.22)
3	The teaching methods used for Orofacial Pain and TMD in the Oral Medicine course were effective.	54 (46.15)	38 (32.48)	25 (21.37)	117 (100)	2.66 (1.17)
4	The explanations provided by instructors of Oral Medicine Course about Orofacial Pain and TMD were clear.	68 (58.12)	30 (25.64)	19 (16.24)	117 (100)	2.41 (1.11)

* Higher scores reflect stronger agreement, while lower scores indicate stronger disagreement.

**Table 4:** Participants’ assessment of the value and relevance to clinical practice of the orofacial pain and TMD course.

	Statement	Disagree *n* (%)	Neutral *n* (%)	Agree *n* (%)	Total *n* (%)	m (SD) *
1	The Oral Medicine course covers the important tips in the topics of Orofacial Pain and TMD.	71 (60.68)	25 (21.37)	21 (17.95)	117 (100)	2.49 (1.19)
2	The content on Orofacial Pain and TMD content are important to my future dental practice.	105 (89.74)	5 (4.27)	7 (5.98)	117 (100)	1.62 (0.97)
3	I perceive the need to increase Orofacial Pain and TMD content in the dental program.	94 (80.34)	14 (11.97)	9 (7.69)	117 (100)	1.79 (1.07)
4	I believe Orofacial Pain and TMD content are important for maintaining licensure in dentistry.	85 (72.65)	24 (20.51)	8 (6.84)	117 (100)	2.06 (1.04)

* Higher scores reflect stronger agreement, while lower scores indicate stronger disagreement.

**Table 5:** Participants’ total scores for course effectiveness and course quality, and course values against gender, sector, study/work area, and previous clinical experiences.

Variable	Total Course Effectiveness	Parametric Test (*p*); Non-Parametric Test (*p*)	Total Course Quality	Parametric Test (*p*); Non-Parametric Test (*p*)	Total Course Value	Parametric Test (*p*); Non-Parametric Test (*p*)
Gender	m ± SD; Median [Q3–Q1]: 95% CL		m ± SD; Median [Q3–Q1]: 95% CL		m ± SD; Median [Q3–Q1]: 95% CL	
Male (n = 59)	24.59 ± 9.20; Median 23 [16–22,25–29]; 95% CI 21.87–27.31	t(115) = −0.24, *p* = 0.812; U = 3430.5, *p* = 0.796	10.32 ± 4.39; Median 9 [7–13]; IQR = 7.0; 95% CI 9.02–11.12	t(115) = −0.54, *p* = 0.591; U = 3345.0, *p* = 0.601	7.97 ± 2.88; Median 8 [6–9]; IQR = 3.0; 95% CI 7.09–8.85	t(115) = 0.06, *p* = 0.952; U = 3564.5, *p* = 0.947
Female (n = 58)	25.00 ± 9.27; Median 25.5 [18–22,25–28]; IQR = 8.25; 95% CI 22.25–27.75		10.74 ± 4.03; Median 11 [8–13]; IQR = 5.25; 95% CI 9.69–11.79		7.93 ± 3.35; Median 7 [6–9]; IQR = 3.0; 95% CI 7.04–8.82	
Sector						
Governmental (n = 90)	24.62 ± 9.11; Median 24 [16–22,25–29]; IQR = 11.0; 95% CI 22.77–26.47	t(115) = −0.35, *p* = 0.736; U = 5744.5, *p* = 0.745	10.38 ± 4.21; Median 10 [8–13]; IQR = 5.0; 95% CI 9.52–11.24	t(115) = −0.66, *p* = 0.513; U = 5804.5, *p* = 0.520	7.84 ± 3.10; Median 7 [6–9]; IQR = 3.0; 95% CI 7.19–8.49	t(115) = −0.65, *p* = 0.523; U = 5758.0, *p* = 0.529
Private (n = 27)	25.30 ± 9.56; Median 25 [19–22,25–29]; IQR = 9.0; 95% CI 21.82–28.78		10.97 ± 4.21; Median 11 [8–13]; IQR = 5.0; 95% CI 9.34–12.60		8.27 ± 3.15; Median 8 [7–10]; IQR = 4.0; 95% CI 7.02–9.52	
Study/work area						
Governmental university (n = 77)	23.86 ± 8.77; Median 23 [16–22,25–28]; IQR = 10.5; 95% CI 21.87–25.85	F(2, 114) = 1.18, *p* = 0.310; H = 0.40, *p* = 0.526	9.96 ± 4.16; Median 9 [7–12]; IQR = 5.5; 95% CI 9.02–10.91	F(2, 114) = 2.12, *p* = 0.125; H = 2.71, *p* = 0.100	7.68 ± 2.99; Median 7 [6–8]; IQR = 2.5; 95% CI 6.99–8.35	F(2, 114) = 0.95, *p* = 0.391; H = 0.27, *p* = 0.600
Governmental workplace (n = 14)	26.36 ± 10.97; Median 26 [17–22,25–37]; IQR = 18.75; 95% CI 20.03–32.69		11.79 ± 4.19; Median 12 [9–14]; IQR = 6.0; 95% CI 9.37–14.21		8.21 ± 3.62; Median 8 [7–9]; IQR = 3.5; 95% CI 6.12–10.30	
Private workplace (n = 26)	26.73 ± 9.37; Median 26.5 [20–22,25–30]; IQR = 8.5; 95% CI 22.94–30.52		11.54 ± 4.15; Median 11 [9–13]; IQR = 4.75; 95% CI 9.86–13.21		8.62 ± 3.18; Median 8.5 [7–10]; IQR = 3.0; 95% CI 7.33–9.89	
Clinical exposure						
Yes (n = 97)	24.86 ± 9.46; Median 25 [16–22,25–29]; IQR = 11.0; 95% CI 22.99–26.73	t(115) = 0.16, *p* = 0.876; U = 948.5, *p* = 0.876	10.61 ± 4.15; Median 10 [8–13]; IQR = 5.0; 95% CI 9.78–11.44	t(115) = 0.44, *p* = 0.659; U = 888.5, *p* = 0.554	8.05 ± 3.35; Median 8 [6–9]; IQR = 3.0; 95% CI 7.38–8.72	t(115) = 0.79, *p* = 0.433; U = 935.0, *p* = 0.798
No (n = 20)	24.50 ± 7.96; Median 24 [18–22,25–28]; IQR = 8.25; 95% CI 20.72–28.28		10.15 ± 4.52; Median 9 [7–14]; IQR = 8.25; 95% CI 8.05–12.25		7.45 ± 1.36; Median 7 [7–9]; IQR = 2.0; 95% CI 6.81–8.09	

Notes: m, mean; SD, standard deviation; CI, confidence interval; Median [IQR], median and interquartile range (Q3–Q1), t, independent-samples *t*-test; F, one-way ANOVA; U, Mann–Whitney U statistic; H, Kruskal–Wallis test statistic. Tests: For gender, sector, and clinical exposure, we used the *t*-test and Mann–Whitney U test (non-parametric). For study/work area, we used ANOVA and the Kruskal–Wallis test (non-parametric). Note: Higher total scores reflect stronger agreement, while lower scores indicate stronger disagreement.

## Data Availability

The original contributions presented in this study are included in the Supplementary Materials.

## Supplementary Materials

The following supporting information can be downloaded at: https://www.mdpi.com/article/10.3390/dj13100465/s1, anonymized raw data file and the modified questionnaire used in the current study.

## Abbreviations

The following abbreviations are used in this manuscript:

OFP	Orofacial pain
TMD	Temporomandibular disorder
OM	Oral Medicine
UQUDENT	Umm Al-Qura University’s Faculty of Dental Medicine
SD	Standard deviation
AAOP	American Academy of Orofacial Pain
IASP	International Association for the Study of Pain
DC/TMD	Diagnostic Criteria for Temporomandibular Disorders
SCFHS	Saudi Commission for Health Specialties
ADA	American Dental Association
IRB	Institutional Review Board
