# Supplementary material for: Orofacial Pain and Temporomandibular Disorders Education at Umm Al-Qura University: Perceptions and Curriculum Improvement Recommendations
Source: Dent J (Basel). 2025 Oct 10;13(10):465. doi: 10.3390/dj13100465 (PMC12562374; doi:10.3390/dj13100465)
Supplement: Supplementary file 1 [file dentistry-13-00465-s001.zip › dentistry-3863677 - file S1. Questionnaire with informed consent .pdf]

Title: **The Perception of Orofacial Pain & TMDs Teaching in the Oral Medicine Course at UQUDENT**

Introduction: You are invited to participate in a research study that will be conducted by Dr. M. Al-Harthy at Umm Al-Qura University's Faculty of Dental Medicine. The purpose of this study is to evaluate how effectively the Oral Medicine course teaches orofacial pain and temporomandibular disorders (TMD), based on graduates and student perspectives. Your feedback will help improve dental education at UQU.

Participation: This survey is aimed at current students (4th year and above) and graduates who have completed the Oral Medicine course in the 4th academic year. Participation involves completing a short questionnaire, which will take approximately 2–3 minutes. Your involvement is entirely voluntary.

Confidentiality: Your responses will remain anonymous. No identifying information will be collected and results will be reported and published for academic purposes without any link to individual participants.

Risks and Benefits: There are no anticipated risks to participating. While there is no direct benefit to you, your input may contribute to enhancing the topics about Orofacial Pain and TMD in in Oral Medicine course for future students.

Consent: By proceeding to complete this questionnaire, you indicate that you have read and understood this information and voluntarily consent to participate.

UQU-IRB: Ethical Approval No. (HAPO-02-K-012-2025-05-2699)

Note: If you have questions or concerns, please contact the PI Dr. M Al-Harthy at: mhharthy@uqu.edu.sa

1. **What is your gender?**
    - Female
    - Male

2. **Age in years?**
    - 20-30
    - 31-40
    - 41-50
    - Above 50

3. **Type of job/ study:**
    - Private sector job/ University
    - Public sector job/ University

4. **What is your current year of study in the Faculty of Dental Medicine?**
    - 4th Year
    - 5th Year
    - 6th Year
    - Intern
    - Graduate

5. **Have you had clinical exposure to orofacial pain or TMD cases as part of your training?**
    - Yes
    - No

6. **The Oral Medicine course covers the important tips in the topics of Orofacial Pain and TMD:**
    - Strongly Disagree
    - Disagree
    - Neutral
    - Agree
    - Strongly Agree

7. **The content on Orofacial Pain and TMD content are important to my future dental practice.**
    - Strongly Disagree
    - Disagree
    - Neutral
    - Agree
    - Strongly Agree

8. **The Orofacial Pain and TMD content improved my dental knowledge.**
    - Strongly Disagree
    - Disagree
    - Neutral
    - Agree
    - Strongly Agree

9. **The Orofacial Pain and TMD content improved my clinical skills.**
    - Strongly Disagree
    - Disagree
    - Neutral
    - Agree
    - Strongly Agree

10. The Orofacial Pain and TMD content improved my clinical decision-making.

- Strongly Disagree
- Disagree
- Neutral
- Agree
- Strongly Agree

11. The Orofacial Pain and TMD content helped me achieve patient satisfaction.

- Strongly Disagree
- Disagree
- Neutral
- Agree
- Strongly Agree

12. The Orofacial Pain and TMD content increased my clinical practice.

- Strongly Disagree
- Disagree
- Neutral
- Agree
- Strongly Agree

13. The Orofacial Pain and TMD content improved the quality of dental care provided to my patients.

- Strongly Disagree
- Disagree
- Neutral
- Agree
- Strongly Agree

14. Overall, how satisfied are you with the teaching of orofacial pain and TMD in the Oral Medicine course?

- Strongly Disagree
- Disagree
- Neutral
- Agree
- Strongly Agree

-

15. The Orofacial Pain and TMD content improved my motivation for learning.

- Strongly Disagree
- Disagree
- Neutral
- Agree
- Strongly Agree

16. The Orofacial Pain and TMD content helped me network with professionals in dentistry.

- Strongly Disagree
- Disagree
- Neutral
- Agree
- Strongly Agree

17. I am satisfied with the quality of the Orofacial Pain and TMD content in the Oral Medicine course.

- Strongly Disagree
- Disagree
- Neutral
- Agree
- Strongly Agree

18. The Orofacial Pain and TMD content was effectively organized.

- Strongly Disagree
- Disagree
- Neutral
- Agree
- Strongly Agree

19. I perceive the need to increase Orofacial Pain and TMD content in the dental program.

- Strongly Disagree
- Disagree
- Neutral
- Agree
- Strongly Agree

20. I believe Orofacial Pain and TMD content are important for maintaining licensure in dentistry.

- Strongly Disagree
- Disagree
- Neutral
- Agree
- Strongly Agree

21. The teaching methods (e.g., lectures, clinical practice, case presentations) used for Orofacial Pain and TMD in the Oral Medicine course were effective:

- Strongly Disagree
- Disagree
- Neutral
- Agree
- Strongly Agree

22. The explanations provided by instructors of Oral Medicine Course about Orofacial Pain and TMD were clear:

- Strongly Disagree
- Disagree
- Neutral
- Agree
- Strongly Agree

23. I am confident to diagnose orofacial pain and TMD after taking the Oral Medicine course.

- Strongly Disagree
- Disagree
- Neutral
- Agree
- Strongly Agree

24. I am confident to manage patients with orofacial pain and TMD after taking the Oral Medicine course.

- Strongly Disagree
- Disagree
- Neutral
- Agree
- Strongly Agree

25. Please feel free to write any suggestions to change or improve how Orofacial Pain and TMD are taught in the Oral Medicine course? (Open text response)

____

____

____
