# Supplementary material for: Orofacial Pain and Temporomandibular Disorders Education at Umm Al-Qura University: Perceptions and Curriculum Improvement Recommendations
Source: Dent J (Basel). 2025 Oct 10;13(10):465. doi: 10.3390/dj13100465 (PMC12562374; doi:10.3390/dj13100465)
Supplement: Supplementary file 1 [file dentistry-13-00465-s001.zip › dentistry-3863677 - table S2. Table of Raw Data.pdf]

	SN	Gender	Age	Sector	Year
1	1.00	2.00	1.00	1.00	4.00
2	2.00	2.00	1.00	1.00	4.00
3	3.00	2.00	1.00	1.00	4.00
4	4.00	2.00	1.00	1.00	4.00
5	5.00	2.00	1.00	1.00	4.00
6	6.00	2.00	1.00	1.00	4.00
7	9.00	2.00	1.00	1.00	4.00
8	10.00	2.00	1.00	1.00	4.00
9	13.00	2.00	1.00	1.00	4.00
10	25.00	2.00	1.00	1.00	4.00
11	26.00	1.00	1.00	1.00	4.00
12	27.00	1.00	1.00	1.00	4.00
13	28.00	1.00	1.00	1.00	4.00
14	29.00	1.00	1.00	1.00	4.00
15	30.00	1.00	1.00	1.00	4.00
16	31.00	1.00	1.00	1.00	4.00
17	34.00	2.00	1.00	1.00	4.00
18	92.00	2.00	1.00	2.00	4.00
19	98.00	2.00	1.00	1.00	4.00
20	110.00	2.00	1.00	1.00	4.00
21	36.00	1.00	1.00	1.00	5.00
22	37.00	1.00	1.00	1.00	5.00
23	38.00	1.00	1.00	1.00	5.00
24	39.00	1.00	1.00	2.00	5.00
25	40.00	1.00	1.00	1.00	5.00
26	41.00	1.00	1.00	2.00	5.00
27	42.00	1.00	1.00	1.00	5.00
28	44.00	1.00	1.00	1.00	5.00
29	53.00	1.00	1.00	1.00	5.00
30	58.00	2.00	1.00	1.00	5.00
31	60.00	2.00	1.00	1.00	5.00
32	61.00	2.00	1.00	1.00	5.00
33	62.00	2.00	1.00	1.00	5.00
34	63.00	2.00	1.00	1.00	5.00
35	84.00	1.00	1.00	2.00	5.00
36	90.00	1.00	1.00	1.00	5.00
37	111.00	2.00	1.00	1.00	5.00
38	11.00	2.00	1.00	1.00	6.00
39	45.00	1.00	1.00	1.00	6.00
40	46.00	1.00	1.00	1.00	6.00
41	47.00	1.00	1.00	1.00	6.00
42	48.00	1.00	1.00	1.00	6.00
43	49.00	1.00	1.00	1.00	6.00
44	50.00	1.00	1.00	1.00	6.00
45	59.00	1.00	1.00	1.00	6.00

	Study_work_area2	Clinical_exp	D1	D2	D3
1	1.00	1.00	2.00	3.00	2.00
2	1.00	1.00	2.00	2.00	2.00
3	1.00	1.00	3.00	3.00	3.00
4	1.00	1.00	1.00	1.00	2.00
5	1.00	1.00	3.00	3.00	3.00
6	1.00	1.00	3.00	2.00	3.00
7	1.00	1.00	2.00	2.00	3.00
8	1.00	1.00	1.00	1.00	1.00
9	1.00	1.00	1.00	1.00	1.00
10	1.00	1.00	2.00	2.00	2.00
11	1.00	2.00	2.00	2.00	2.00
12	1.00	1.00	2.00	1.00	4.00
13	1.00	1.00	3.00	3.00	3.00
14	1.00	1.00	1.00	1.00	1.00
15	1.00	1.00	1.00	1.00	1.00
16	1.00	2.00	1.00	2.00	2.00
17	1.00	1.00	2.00	2.00	2.00
18	1.00	1.00	1.00	1.00	1.00
19	1.00	2.00	2.00	2.00	2.00
20	1.00	1.00	2.00	3.00	3.00
21	1.00	1.00	3.00	3.00	3.00
22	1.00	1.00	1.00	2.00	2.00
23	1.00	1.00	2.00	2.00	3.00
24	1.00	1.00	1.00	1.00	1.00
25	1.00	2.00	2.00	3.00	2.00
26	1.00	1.00	2.00	2.00	3.00
27	1.00	1.00	3.00	4.00	2.00
28	1.00	1.00	2.00	3.00	2.00
29	1.00	2.00	2.00	3.00	3.00
30	1.00	1.00	2.00	3.00	3.00
31	1.00	2.00	1.00	1.00	3.00
32	1.00	1.00	1.00	2.00	2.00
33	1.00	2.00	2.00	1.00	1.00
34	1.00	1.00	3.00	3.00	4.00
35	1.00	1.00	1.00	1.00	1.00
36	1.00	1.00	5.00	5.00	5.00
37	1.00	1.00	2.00	2.00	2.00
38	1.00	1.00	3.00	2.00	2.00
39	1.00	1.00	1.00	1.00	2.00
40	1.00	1.00	2.00	3.00	1.00
41	1.00	1.00	1.00	1.00	1.00
42	1.00	1.00	3.00	2.00	3.00
43	1.00	2.00	1.00	2.00	2.00
44	1.00	1.00	3.00	3.00	2.00
45	1.00	1.00	1.00	1.00	1.00

	D4	D5	D6	D7	D8	D9	D10
1	3.00	4.00	1.00	3.00	2.00	1.00	2.00
2	2.00	2.00	2.00	2.00	2.00	2.00	2.00
3	3.00	3.00	3.00	3.00	2.00	4.00	4.00
4	2.00	2.00	1.00	2.00	1.00	1.00	1.00
5	2.00	2.00	2.00	3.00	4.00	2.00	2.00
6	3.00	3.00	3.00	3.00	3.00	3.00	3.00
7	2.00	3.00	1.00	1.00	2.00	4.00	4.00
8	1.00	1.00	1.00	1.00	1.00	1.00	1.00
9	1.00	1.00	1.00	1.00	1.00	3.00	3.00
10	2.00	3.00	2.00	2.00	1.00	3.00	2.00
11	3.00	3.00	2.00	3.00	3.00	3.00	3.00
12	2.00	3.00	3.00	2.00	2.00	4.00	3.00
13	3.00	3.00	3.00	4.00	3.00	1.00	1.00
14	1.00	1.00	1.00	1.00	1.00	3.00	3.00
15	2.00	1.00	2.00	2.00	2.00	2.00	3.00
16	2.00	1.00	1.00	1.00	1.00	2.00	2.00
17	1.00	1.00	1.00	1.00	2.00	1.00	2.00
18	1.00	3.00	1.00	3.00	1.00	3.00	3.00
19	3.00	2.00	2.00	3.00	2.00	2.00	3.00
20	3.00	2.00	2.00	2.00	2.00	3.00	2.00
21	3.00	3.00	3.00	3.00	3.00	3.00	3.00
22	2.00	1.00	1.00	2.00	1.00	3.00	2.00
23	3.00	3.00	3.00	3.00	3.00	5.00	5.00
24	1.00	1.00	1.00	1.00	1.00	1.00	3.00
25	2.00	2.00	2.00	2.00	2.00	2.00	2.00
26	2.00	2.00	2.00	1.00	1.00	4.00	2.00
27	2.00	4.00	4.00	4.00	4.00	1.00	1.00
28	2.00	4.00	3.00	3.00	3.00	2.00	3.00
29	3.00	1.00	1.00	1.00	3.00	1.00	1.00
30	2.00	3.00	3.00	3.00	2.00	4.00	3.00
31	3.00	3.00	3.00	2.00	4.00	3.00	4.00
32	3.00	3.00	2.00	2.00	2.00	1.00	2.00
33	1.00	1.00	1.00	1.00	1.00	1.00	1.00
34	4.00	4.00	4.00	4.00	4.00	4.00	4.00
35	1.00	1.00	1.00	1.00	1.00	3.00	2.00
36	5.00	5.00	5.00	5.00	5.00	5.00	5.00
37	2.00	2.00	2.00	2.00	2.00	5.00	5.00
38	3.00	2.00	2.00	3.00	2.00	4.00	4.00
39	1.00	1.00	2.00	2.00	1.00	4.00	1.00
40	3.00	1.00	1.00	3.00	1.00	1.00	2.00
41	1.00	1.00	1.00	1.00	1.00	1.00	1.00
42	1.00	2.00	2.00	2.00	1.00	2.00	1.00
43	3.00	2.00	2.00	2.00	2.00	1.00	1.00
44	3.00	4.00	2.00	2.00	2.00	4.00	4.00
45	1.00	1.00	2.00	2.00	2.00	2.00	2.00

	E1	E2	E3	E4	F1	F2	F3
1	2.00	3.00	2.00	2.00	3.00	2.00	1.00
2	2.00	2.00	3.00	3.00	2.00	2.00	2.00
3	4.00	3.00	4.00	4.00	3.00	1.00	1.00
4	3.00	2.00	3.00	1.00	1.00	1.00	3.00
5	3.00	3.00	3.00	2.00	3.00	1.00	2.00
6	3.00	3.00	3.00	3.00	2.00	2.00	3.00
7	4.00	4.00	3.00	3.00	3.00	1.00	1.00
8	1.00	1.00	1.00	1.00	1.00	1.00	1.00
9	4.00	1.00	1.00	1.00	5.00	1.00	1.00
10	2.00	3.00	2.00	2.00	2.00	1.00	2.00
11	2.00	3.00	2.00	1.00	2.00	2.00	1.00
12	5.00	3.00	3.00	3.00	2.00	1.00	1.00
13	1.00	1.00	3.00	1.00	1.00	1.00	1.00
14	1.00	1.00	1.00	2.00	1.00	1.00	2.00
15	1.00	1.00	1.00	1.00	1.00	1.00	1.00
16	2.00	2.00	3.00	2.00	2.00	1.00	2.00
17	2.00	2.00	2.00	2.00	3.00	1.00	1.00
18	3.00	3.00	2.00	1.00	2.00	1.00	1.00
19	3.00	3.00	2.00	2.00	3.00	1.00	2.00
20	3.00	3.00	3.00	3.00	3.00	2.00	1.00
21	3.00	3.00	3.00	3.00	4.00	1.00	1.00
22	1.00	2.00	3.00	2.00	2.00	2.00	3.00
23	4.00	4.00	4.00	4.00	2.00	2.00	2.00
24	1.00	1.00	2.00	1.00	1.00	1.00	1.00
25	2.00	2.00	2.00	2.00	2.00	2.00	2.00
26	4.00	1.00	3.00	2.00	4.00	3.00	1.00
27	3.00	1.00	1.00	1.00	2.00	2.00	1.00
28	2.00	2.00	3.00	2.00	2.00	1.00	3.00
29	3.00	1.00	1.00	1.00	2.00	3.00	1.00
30	3.00	3.00	3.00	3.00	2.00	1.00	1.00
31	1.00	1.00	1.00	1.00	1.00	1.00	4.00
32	2.00	2.00	1.00	1.00	2.00	1.00	1.00
33	1.00	1.00	1.00	1.00	3.00	1.00	1.00
34	3.00	4.00	4.00	4.00	4.00	1.00	2.00
35	2.00	1.00	1.00	1.00	2.00	1.00	1.00
36	5.00	5.00	5.00	5.00	5.00	5.00	5.00
37	3.00	3.00	4.00	4.00	3.00	2.00	1.00
38	4.00	3.00	2.00	3.00	2.00	1.00	2.00
39	1.00	1.00	1.00	1.00	2.00	1.00	5.00
40	3.00	2.00	2.00	4.00	2.00	3.00	1.00
41	1.00	1.00	1.00	1.00	1.00	1.00	1.00
42	2.00	2.00	2.00	2.00	2.00	2.00	1.00
43	2.00	2.00	1.00	1.00	5.00	1.00	2.00
44	2.00	2.00	2.00	2.00	3.00	2.00	1.00
45	2.00	2.00	1.00	1.00	2.00	1.00	2.00

	F4	Total_effectivness	Total_quality	Total_value
1	1.00	23.00	9.00	7.00
2	2.00	20.00	10.00	8.00
3	3.00	31.00	15.00	8.00
4	3.00	14.00	9.00	8.00
5	3.00	26.00	11.00	9.00
6	3.00	29.00	12.00	10.00
7	1.00	24.00	14.00	6.00
8	1.00	10.00	4.00	4.00
9	1.00	14.00	7.00	8.00
10	2.00	21.00	9.00	7.00
11	2.00	26.00	8.00	7.00
12	1.00	26.00	14.00	5.00
13	1.00	27.00	6.00	4.00
14	2.00	14.00	5.00	6.00
15	1.00	17.00	4.00	4.00
16	2.00	15.00	9.00	7.00
17	1.00	15.00	8.00	6.00
18	1.00	18.00	9.00	5.00
19	2.00	23.00	10.00	8.00
20	2.00	24.00	12.00	8.00
21	1.00	30.00	12.00	7.00
22	3.00	17.00	8.00	10.00
23	2.00	32.00	16.00	8.00
24	2.00	12.00	5.00	5.00
25	2.00	21.00	8.00	8.00
26	1.00	21.00	10.00	9.00
27	1.00	29.00	6.00	6.00
28	2.00	27.00	9.00	8.00
29	1.00	19.00	6.00	7.00
30	1.00	28.00	12.00	5.00
31	3.00	27.00	4.00	9.00
32	1.00	20.00	6.00	5.00
33	1.00	11.00	4.00	6.00
34	2.00	38.00	15.00	9.00
35	1.00	13.00	5.00	5.00
36	5.00	50.00	20.00	20.00
37	1.00	26.00	14.00	7.00
38	2.00	27.00	12.00	7.00
39	2.00	16.00	4.00	10.00
40	2.00	18.00	11.00	8.00
41	1.00	10.00	4.00	4.00
42	3.00	19.00	8.00	8.00
43	1.00	18.00	6.00	9.00
44	2.00	29.00	8.00	8.00
45	2.00	15.00	6.00	7.00

	SN	Gender	Age	Sector	Year
46	64.00	1.00	1.00	1.00	6.00
47	65.00	1.00	1.00	1.00	6.00
48	66.00	1.00	1.00	1.00	6.00
49	67.00	1.00	1.00	1.00	6.00
50	68.00	1.00	1.00	1.00	6.00
51	72.00	1.00	1.00	1.00	6.00
52	78.00	2.00	1.00	1.00	6.00
53	81.00	2.00	1.00	1.00	6.00
54	87.00	2.00	1.00	1.00	6.00
55	96.00	2.00	1.00	1.00	6.00
56	106.00	1.00	1.00	1.00	6.00
57	112.00	2.00	1.00	1.00	6.00
58	8.00	2.00	1.00	1.00	7.00
59	19.00	1.00	1.00	1.00	7.00
60	22.00	1.00	1.00	1.00	7.00
61	43.00	2.00	1.00	1.00	7.00
62	51.00	2.00	1.00	1.00	7.00
63	52.00	1.00	1.00	1.00	7.00
64	55.00	1.00	1.00	1.00	7.00
65	69.00	1.00	1.00	1.00	7.00
66	73.00	1.00	1.00	1.00	7.00
67	83.00	2.00	1.00	1.00	7.00
68	93.00	2.00	1.00	1.00	7.00
69	94.00	1.00	1.00	1.00	7.00
70	95.00	1.00	1.00	1.00	7.00
71	101.00	1.00	1.00	1.00	7.00
72	104.00	2.00	1.00	1.00	7.00
73	105.00	1.00	1.00	1.00	7.00
74	107.00	2.00	1.00	1.00	7.00
75	113.00	2.00	1.00	1.00	7.00
76	114.00	1.00	1.00	1.00	7.00
77	117.00	1.00	1.00	1.00	7.00
78	7.00	2.00	1.00	2.00	8.00
79	12.00	1.00	1.00	1.00	8.00
80	14.00	1.00	1.00	2.00	8.00
81	15.00	2.00	1.00	1.00	8.00
82	16.00	1.00	1.00	1.00	8.00
83	17.00	2.00	1.00	2.00	8.00
84	18.00	2.00	1.00	2.00	8.00
85	20.00	1.00	1.00	2.00	8.00
86	21.00	2.00	1.00	1.00	8.00
87	23.00	1.00	1.00	2.00	8.00
88	24.00	1.00	1.00	1.00	8.00
89	32.00	2.00	1.00	2.00	8.00
90	33.00	1.00	1.00	1.00	8.00

	Study_work_area2	Clinical_exp	D1	D2	D3
46	1.00	1.00	2.00	2.00	2.00
47	1.00	1.00	2.00	3.00	3.00
48	1.00	1.00	1.00	1.00	1.00
49	1.00	1.00	2.00	2.00	2.00
50	1.00	1.00	1.00	1.00	1.00
51	1.00	2.00	2.00	2.00	2.00
52	1.00	2.00	1.00	1.00	1.00
53	1.00	1.00	3.00	2.00	2.00
54	1.00	1.00	2.00	2.00	3.00
55	1.00	1.00	2.00	3.00	2.00
56	1.00	1.00	2.00	2.00	3.00
57	1.00	2.00	1.00	1.00	3.00
58	1.00	1.00	3.00	3.00	3.00
59	1.00	1.00	2.00	2.00	4.00
60	1.00	1.00	2.00	2.00	2.00
61	1.00	1.00	2.00	1.00	3.00
62	1.00	1.00	2.00	3.00	2.00
63	1.00	1.00	2.00	4.00	3.00
64	1.00	2.00	2.00	2.00	2.00
65	1.00	1.00	3.00	3.00	4.00
66	1.00	1.00	1.00	1.00	1.00
67	1.00	1.00	3.00	3.00	3.00
68	1.00	2.00	3.00	3.00	2.00
69	1.00	2.00	2.00	4.00	5.00
70	1.00	1.00	1.00	4.00	2.00
71	1.00	1.00	2.00	2.00	2.00
72	1.00	1.00	5.00	5.00	5.00
73	1.00	1.00	3.00	3.00	3.00
74	1.00	1.00	5.00	5.00	5.00
75	1.00	1.00	1.00	2.00	3.00
76	1.00	1.00	2.00	2.00	1.00
77	1.00	1.00	1.00	2.00	2.00
78	4.00	1.00	2.00	2.00	3.00
79	3.00	1.00	1.00	1.00	1.00
80	4.00	1.00	1.00	2.00	2.00
81	3.00	2.00	3.00	3.00	4.00
82	3.00	1.00	3.00	2.00	3.00
83	4.00	2.00	1.00	2.00	2.00
84	4.00	2.00	3.00	3.00	4.00
85	4.00	1.00	2.00	5.00	2.00
86	3.00	1.00	2.00	2.00	2.00
87	4.00	2.00	3.00	3.00	3.00
88	3.00	1.00	4.00	4.00	4.00
89	4.00	1.00	2.00	2.00	3.00
90	3.00	1.00	4.00	4.00	4.00

	D4	D5	D6	D7	D8	D9	D10
46	3.00	2.00	2.00	2.00	2.00	3.00	2.00
47	2.00	1.00	2.00	3.00	2.00	4.00	4.00
48	1.00	1.00	1.00	1.00	1.00	1.00	1.00
49	2.00	2.00	3.00	2.00	2.00	3.00	3.00
50	1.00	2.00	1.00	1.00	1.00	1.00	1.00
51	2.00	2.00	3.00	1.00	3.00	2.00	2.00
52	1.00	1.00	1.00	1.00	1.00	2.00	3.00
53	3.00	2.00	2.00	3.00	1.00	3.00	3.00
54	3.00	2.00	2.00	2.00	2.00	2.00	2.00
55	2.00	4.00	1.00	2.00	4.00	4.00	2.00
56	3.00	3.00	2.00	3.00	3.00	3.00	3.00
57	3.00	3.00	3.00	3.00	3.00	3.00	3.00
58	3.00	3.00	3.00	3.00	3.00	5.00	5.00
59	3.00	3.00	3.00	3.00	2.00	4.00	4.00
60	4.00	2.00	2.00	2.00	2.00	4.00	4.00
61	2.00	2.00	3.00	4.00	2.00	2.00	2.00
62	3.00	4.00	3.00	3.00	3.00	5.00	5.00
63	5.00	3.00	3.00	2.00	2.00	4.00	3.00
64	2.00	2.00	2.00	2.00	2.00	4.00	2.00
65	4.00	3.00	1.00	3.00	2.00	2.00	3.00
66	1.00	2.00	1.00	1.00	3.00	2.00	2.00
67	4.00	4.00	3.00	3.00	5.00	5.00	4.00
68	3.00	3.00	1.00	2.00	3.00	4.00	3.00
69	5.00	4.00	4.00	4.00	3.00	5.00	5.00
70	4.00	2.00	2.00	1.00	2.00	2.00	2.00
71	3.00	4.00	4.00	2.00	3.00	3.00	3.00
72	5.00	5.00	5.00	5.00	5.00	5.00	5.00
73	3.00	3.00	3.00	3.00	2.00	5.00	5.00
74	5.00	5.00	5.00	5.00	5.00	5.00	5.00
75	3.00	4.00	4.00	3.00	2.00	4.00	4.00
76	3.00	2.00	1.00	1.00	2.00	1.00	1.00
77	2.00	2.00	2.00	2.00	2.00	3.00	2.00
78	2.00	3.00	3.00	3.00	3.00	4.00	3.00
79	2.00	2.00	1.00	2.00	3.00	2.00	1.00
80	2.00	3.00	2.00	1.00	4.00	3.00	3.00
81	4.00	4.00	3.00	4.00	4.00	4.00	4.00
82	3.00	3.00	2.00	2.00	2.00	3.00	3.00
83	3.00	3.00	3.00	2.00	3.00	3.00	3.00
84	4.00	4.00	4.00	4.00	4.00	4.00	5.00
85	1.00	4.00	2.00	3.00	4.00	4.00	2.00
86	4.00	2.00	2.00	2.00	2.00	4.00	4.00
87	3.00	3.00	3.00	2.00	3.00	3.00	3.00
88	4.00	4.00	4.00	2.00	3.00	4.00	4.00
89	2.00	5.00	2.00	3.00	2.00	5.00	5.00
90	4.00	4.00	4.00	4.00	2.00	4.00	4.00

	E1	E2	E3	E4	F1	F2	F3
46	2.00	2.00	3.00	2.00	1.00	1.00	2.00
47	1.00	1.00	2.00	2.00	2.00	2.00	1.00
48	1.00	1.00	1.00	1.00	1.00	1.00	1.00
49	3.00	3.00	3.00	3.00	3.00	2.00	1.00
50	1.00	1.00	1.00	2.00	2.00	1.00	1.00
51	3.00	2.00	2.00	2.00	1.00	2.00	2.00
52	3.00	3.00	2.00	1.00	1.00	1.00	3.00
53	3.00	2.00	2.00	3.00	2.00	2.00	3.00
54	2.00	2.00	2.00	2.00	2.00	1.00	2.00
55	2.00	2.00	4.00	3.00	2.00	1.00	4.00
56	3.00	2.00	2.00	3.00	2.00	1.00	2.00
57	1.00	1.00	1.00	1.00	1.00	1.00	1.00
58	3.00	3.00	4.00	4.00	3.00	1.00	3.00
59	4.00	3.00	2.00	2.00	2.00	2.00	1.00
60	2.00	4.00	2.00	2.00	2.00	2.00	1.00
61	2.00	2.00	3.00	2.00	2.00	2.00	1.00
62	5.00	4.00	4.00	3.00	4.00	2.00	2.00
63	3.00	4.00	3.00	3.00	2.00	2.00	1.00
64	5.00	5.00	4.00	3.00	4.00	2.00	1.00
65	3.00	2.00	2.00	2.00	3.00	1.00	2.00
66	3.00	3.00	2.00	3.00	2.00	1.00	1.00
67	4.00	3.00	3.00	2.00	4.00	1.00	1.00
68	4.00	4.00	5.00	2.00	2.00	2.00	2.00
69	4.00	5.00	5.00	5.00	3.00	2.00	1.00
70	2.00	3.00	2.00	2.00	2.00	2.00	3.00
71	4.00	4.00	3.00	3.00	2.00	2.00	2.00
72	5.00	5.00	5.00	5.00	5.00	5.00	5.00
73	4.00	4.00	4.00	3.00	2.00	1.00	1.00
74	5.00	5.00	5.00	5.00	5.00	5.00	5.00
75	4.00	4.00	4.00	4.00	2.00	1.00	2.00
76	3.00	3.00	1.00	1.00	2.00	1.00	3.00
77	2.00	2.00	2.00	2.00	2.00	1.00	2.00
78	4.00	3.00	3.00	3.00	3.00	1.00	1.00
79	1.00	1.00	2.00	2.00	1.00	1.00	1.00
80	3.00	3.00	3.00	3.00	3.00	3.00	1.00
81	4.00	4.00	3.00	3.00	5.00	1.00	1.00
82	3.00	3.00	3.00	3.00	3.00	2.00	2.00
83	3.00	3.00	3.00	2.00	2.00	2.00	1.00
84	4.00	4.00	4.00	4.00	3.00	1.00	2.00
85	2.00	4.00	2.00	1.00	1.00	2.00	2.00
86	3.00	4.00	3.00	2.00	3.00	1.00	1.00
87	4.00	4.00	4.00	3.00	2.00	1.00	1.00
88	4.00	4.00	4.00	4.00	4.00	4.00	1.00
89	5.00	4.00	5.00	4.00	5.00	4.00	1.00
90	4.00	5.00	4.00	4.00	5.00	1.00	1.00

	F4	Total_effectivness	Total_quality	Total_value
46	2.00	22.00	9.00	6.00
47	2.00	26.00	6.00	7.00
48	1.00	10.00	4.00	4.00
49	2.00	23.00	12.00	8.00
50	1.00	11.00	5.00	5.00
51	3.00	21.00	9.00	8.00
52	1.00	13.00	9.00	6.00
53	2.00	24.00	10.00	9.00
54	2.00	22.00	8.00	7.00
55	2.00	26.00	11.00	9.00
56	2.00	27.00	10.00	7.00
57	1.00	26.00	4.00	4.00
58	3.00	34.00	14.00	10.00
59	2.00	30.00	11.00	7.00
60	2.00	26.00	10.00	7.00
61	1.00	23.00	9.00	6.00
62	2.00	33.00	16.00	10.00
63	3.00	31.00	13.00	8.00
64	2.00	22.00	17.00	9.00
65	2.00	28.00	9.00	8.00
66	1.00	15.00	11.00	5.00
67	2.00	37.00	12.00	8.00
68	3.00	27.00	15.00	9.00
69	1.00	41.00	19.00	7.00
70	3.00	22.00	9.00	10.00
71	2.00	28.00	14.00	8.00
72	5.00	50.00	20.00	20.00
73	2.00	33.00	15.00	6.00
74	5.00	50.00	20.00	20.00
75	2.00	30.00	16.00	7.00
76	3.00	16.00	8.00	9.00
77	2.00	20.00	8.00	7.00
78	2.00	28.00	13.00	7.00
79	2.00	16.00	6.00	5.00
80	2.00	23.00	12.00	9.00
81	2.00	37.00	14.00	9.00
82	3.00	26.00	12.00	10.00
83	2.00	25.00	11.00	7.00
84	3.00	39.00	16.00	9.00
85	4.00	29.00	9.00	9.00
86	2.00	26.00	12.00	7.00
87	2.00	29.00	15.00	6.00
88	3.00	37.00	16.00	12.00
89	1.00	31.00	18.00	11.00
90	1.00	38.00	17.00	8.00

	SN	Gender	Age	Sector	Year
91	35.00	1.00	1.00	2.00	8.00
92	54.00	2.00	1.00	2.00	8.00
93	56.00	2.00	1.00	1.00	8.00
94	57.00	2.00	1.00	2.00	8.00
95	70.00	1.00	1.00	2.00	8.00
96	71.00	1.00	1.00	2.00	8.00
97	74.00	2.00	1.00	1.00	8.00
98	75.00	2.00	1.00	1.00	8.00
99	76.00	2.00	1.00	2.00	8.00
100	77.00	2.00	1.00	2.00	8.00
101	79.00	2.00	1.00	2.00	8.00
102	80.00	2.00	1.00	2.00	8.00
103	82.00	1.00	2.00	1.00	8.00
104	85.00	2.00	1.00	2.00	8.00
105	86.00	1.00	1.00	1.00	8.00
106	88.00	2.00	1.00	1.00	8.00
107	89.00	1.00	1.00	2.00	8.00
108	91.00	2.00	1.00	2.00	8.00
109	97.00	1.00	1.00	2.00	8.00
110	99.00	2.00	1.00	1.00	8.00
111	100.00	2.00	1.00	2.00	8.00
112	102.00	2.00	1.00	2.00	8.00
113	103.00	1.00	1.00	1.00	8.00
114	108.00	2.00	1.00	2.00	8.00
115	109.00	1.00	1.00	2.00	8.00
116	115.00	2.00	1.00	2.00	8.00
117	116.00	2.00	1.00	2.00	8.00

	Study_work_area2	Clinical_exp	D1	D2	D3
91	4.00	1.00	3.00	4.00	4.00
92	4.00	2.00	2.00	3.00	3.00
93	3.00	1.00	1.00	1.00	1.00
94	4.00	1.00	1.00	1.00	1.00
95	4.00	1.00	2.00	3.00	3.00
96	4.00	1.00	2.00	2.00	3.00
97	3.00	1.00	1.00	1.00	3.00
98	3.00	1.00	1.00	2.00	3.00
99	4.00	1.00	5.00	5.00	5.00
100	4.00	1.00	1.00	2.00	2.00
101	4.00	1.00	3.00	2.00	4.00
102	4.00	1.00	1.00	2.00	2.00
103	3.00	1.00	2.00	2.00	2.00
104	4.00	1.00	4.00	2.00	1.00
105	3.00	1.00	1.00	1.00	2.00
106	3.00	1.00	1.00	2.00	1.00
107	4.00	1.00	5.00	5.00	5.00
108	4.00	1.00	1.00	1.00	2.00
109	4.00	1.00	2.00	2.00	3.00
110	3.00	1.00	3.00	2.00	3.00
111	4.00	1.00	3.00	3.00	3.00
112	4.00	1.00	2.00	2.00	2.00
113	3.00	1.00	5.00	5.00	5.00
114	4.00	1.00	2.00	2.00	2.00
115	4.00	1.00	2.00	3.00	2.00
116	4.00	1.00	2.00	2.00	2.00
117	4.00	2.00	1.00	2.00	2.00

	D4	D5	D6	D7	D8	D9	D10
91	3.00	4.00	4.00	2.00	3.00	2.00	3.00
92	3.00	3.00	3.00	2.00	3.00	3.00	3.00
93	1.00	1.00	1.00	1.00	1.00	1.00	2.00
94	1.00	1.00	1.00	1.00	1.00	1.00	1.00
95	3.00	2.00	3.00	2.00	3.00	2.00	2.00
96	3.00	3.00	3.00	2.00	2.00	4.00	4.00
97	3.00	4.00	3.00	2.00	1.00	4.00	4.00
98	3.00	2.00	1.00	2.00	2.00	3.00	3.00
99	5.00	5.00	5.00	5.00	5.00	5.00	5.00
100	2.00	1.00	2.00	2.00	3.00	1.00	1.00
101	3.00	4.00	2.00	3.00	2.00	2.00	2.00
102	2.00	2.00	2.00	2.00	1.00	1.00	1.00
103	2.00	2.00	2.00	2.00	2.00	3.00	3.00
104	2.00	2.00	4.00	1.00	1.00	2.00	2.00
105	2.00	2.00	2.00	1.00	3.00	3.00	2.00
106	1.00	1.00	1.00	1.00	1.00	2.00	1.00
107	5.00	5.00	5.00	5.00	5.00	5.00	5.00
108	1.00	1.00	1.00	1.00	1.00	1.00	1.00
109	2.00	3.00	3.00	3.00	3.00	5.00	5.00
110	3.00	3.00	3.00	3.00	2.00	3.00	2.00
111	3.00	4.00	3.00	3.00	2.00	3.00	3.00
112	3.00	2.00	2.00	2.00	3.00	2.00	3.00
113	5.00	5.00	5.00	5.00	5.00	5.00	5.00
114	2.00	3.00	2.00	1.00	1.00	3.00	3.00
115	2.00	4.00	3.00	1.00	2.00	2.00	2.00
116	3.00	2.00	3.00	3.00	3.00	3.00	3.00
117	3.00	3.00	2.00	2.00	2.00	2.00	3.00

	E1	E2	E3	E4	F1	F2	F3
91	4.00	3.00	3.00	3.00	4.00	3.00	1.00
92	2.00	2.00	3.00	1.00	2.00	1.00	1.00
93	1.00	1.00	1.00	2.00	1.00	1.00	2.00
94	1.00	1.00	1.00	1.00	1.00	1.00	3.00
95	3.00	2.00	2.00	2.00	3.00	1.00	3.00
96	4.00	4.00	3.00	2.00	4.00	1.00	1.00
97	2.00	2.00	4.00	3.00	1.00	1.00	1.00
98	2.00	2.00	3.00	2.00	3.00	1.00	2.00
99	5.00	5.00	5.00	5.00	5.00	5.00	5.00
100	3.00	4.00	2.00	2.00	3.00	1.00	1.00
101	3.00	2.00	3.00	3.00	3.00	2.00	2.00
102	2.00	2.00	2.00	2.00	1.00	1.00	1.00
103	4.00	3.00	3.00	2.00	3.00	2.00	1.00
104	1.00	1.00	1.00	4.00	1.00	1.00	4.00
105	4.00	3.00	3.00	2.00	2.00	2.00	2.00
106	1.00	1.00	3.00	2.00	1.00	1.00	1.00
107	5.00	5.00	5.00	3.00	5.00	2.00	1.00
108	1.00	1.00	2.00	1.00	1.00	1.00	1.00
109	5.00	5.00	5.00	4.00	5.00	1.00	1.00
110	4.00	3.00	3.00	2.00	3.00	2.00	2.00
111	3.00	3.00	3.00	2.00	2.00	2.00	3.00
112	2.00	2.00	2.00	2.00	2.00	1.00	2.00
113	5.00	5.00	5.00	5.00	5.00	5.00	3.00
114	2.00	2.00	3.00	3.00	2.00	1.00	1.00
115	2.00	2.00	2.00	3.00	2.00	2.00	4.00
116	3.00	3.00	2.00	3.00	2.00	1.00	3.00
117	3.00	3.00	3.00	2.00	2.00	1.00	2.00

	F4	Total_effectivness	Total_quality	Total_value
91	3.00	32.00	13.00	11.00
92	3.00	28.00	8.00	7.00
93	2.00	11.00	5.00	6.00
94	1.00	10.00	4.00	6.00
95	3.00	25.00	9.00	10.00
96	1.00	28.00	13.00	7.00
97	1.00	26.00	11.00	4.00
98	1.00	22.00	9.00	7.00
99	5.00	50.00	20.00	20.00
100	1.00	17.00	11.00	6.00
101	3.00	27.00	11.00	10.00
102	1.00	16.00	8.00	4.00
103	3.00	22.00	12.00	9.00
104	2.00	21.00	7.00	8.00
105	1.00	19.00	12.00	7.00
106	1.00	12.00	7.00	4.00
107	1.00	50.00	18.00	9.00
108	1.00	11.00	5.00	4.00
109	5.00	31.00	19.00	12.00
110	2.00	27.00	12.00	9.00
111	3.00	30.00	11.00	10.00
112	2.00	23.00	8.00	7.00
113	5.00	50.00	20.00	18.00
114	3.00	21.00	10.00	7.00
115	4.00	23.00	9.00	12.00
116	3.00	26.00	11.00	9.00
117	2.00	22.00	11.00	7.00
